# Patient-specific implants for maxillofacial defects: challenges and solutions

**DOI:** 10.1186/s40902-020-00262-7

**Published:** 2020-05-20

**Authors:** Nasser Alasseri, Ahmed Alasraj

**Affiliations:** 1 Prince Sultan Military Medical City, Riyadh, Saudi Arabia; 2 Ministry of Health, Riyadh, Saudi Arabia

## Abstract

**Background:**

Reconstructing maxillofacial defects is quite challenging for most surgeons due to the region’s complex anatomy and cosmetic and functional effects on patients. The use of pre-made alloplastic implants and autogenous grafts is often associated with resorption, infection, and displacement. Recent technological advances have led to the use of custom computer-designed patient-specific implants (PSIs) in reconstructive surgery. This study describes our experience with PSI, details the complications we faced, how to overcome them, and finally, evaluates patient satisfaction.

**Case presentation:**

Six patients underwent reconstruction of various maxillofacial defects arising due to different etiologies using PSI. A combined total of 10 implants was used. PEEK was used to fabricate 8, while titanium was used to fabricate 2. No complications were seen in any patient both immediately post-op and in subsequent follow-ups. All patients reported a high level of satisfaction with the final result both functionally and cosmetically.

**Conclusion:**

The use of computer-designed PSI enables a more accurate reconstruction of maxillofacial defects, eliminating the usual complications seen in preformed implants and resulting in higher patient satisfaction. Its main drawback is its high cost.

## Background

The surgical repair and reconstruction of maxillofacial defects, both congenital and acquired, are challenging even for the most seasoned surgeons. This is attributed to the complex anatomy, patient expectations, and defect uniqueness [1].

From a functional and esthetic view, it is imperative for surgeons to accurately restore the defect in a way that will ensure patient satisfaction and well-being. Autogenous grafts are still considered the gold standard for reconstruction by many [2–4]. However, they are often associated with an unpredictable resorption and donor site morbidity rates [5].

The advent of additive manufacturing, 3–dimensional (3D) printing, and the recent advances in those technologies has positively influenced the biomedical field, leading to the utilization of patient-specific implants (PSIs) in the surgical repair of maxillofacial defects [6].

Advanced imaging modalities, such as CT, work with AM technologies to fabricate PSIs that are unique to each defect [7]. The use of PSI offers higher accuracy, better site adaptation, and shortened operating time compared to pre-bent or pre-made implants [8].

Here, we report a series of 6 patients whose maxillofacial defects were surgically reconstructed using computer-designed PSI.

## Materials and methods

Six patients underwent a total of 10 PSIs (8 polyetheretherketones [PEEKs], 2 titaniums) between 2017 and 2018 at the oral and maxillofacial surgery department of Prince Sultan Military Medical City in Riyadh, Saudi Arabia. None of the patients had comorbidities. They all underwent preoperative CT scans 1mm thickness) that were sent to the manufacturer (KLS Martin Group, Germany) through IPS Gate. Treatment planning was carried out using KLS Martin’s Case Designer. The data was then entered into the Geomagic’s Freeform 3D designing software by the manufacturer and the healthy, unaffected side was mirrored for design of the final implant. The surgeon and the manufacturer’s engineer met online to discuss the design and any needed adjustments. The final design was then sent to the surgeon for approval. The final implant was created using a rapid prototyping machine and sent to the hospital and sterilized preoperatively. Patients were operated on using the implants. Finally, they were followed up regularly.

All procedures were performed with the patients under general anesthesia. All patients received an intraoperative dose of intravenous Augmentin 1.2 g. A number of different surgical approaches were used intra- and postoperatively depending on defect size and location. The PSI was checked for fit and contour prior to fixation. Any needed adjustments were made intraoperatively. Final PSI fixation was achieved using 1.5 to 2.0 mm screws. Patients received 2 doses of intravenous Augmentin postoperatively as inpatients. A 5–day oral regimen of Augmentin 1 g was prescribed upon discharge.

## Results

Six patients (3 females and 3 males) underwent PSI maxillofacial reconstruction arising from varying defects and etiologies including Parry-Romberg syndrome (in 1), hemifacial microsomia (in 1), post-bilateral sagittal split osteotomy (in 1), post-craniotomy (in 1), post-free flap reconstruction (in 1), and post-traumatic secondary deformity (in 1). The defects were located in various areas including the cranium (in 1), frontal bone (in 1), zygomatic bone (in 2), nasal bone (in 1), maxillary bone (in 1), orbital bone (in 1), and mandible (in 2). All patients underwent delayed defect reconstruction. A total of 10 custom-made PSIs (8 PEEKs, 2 titaniums) were created. Some cases required minimal adjustment, if any, of the PSI prior to final fixation.

## Case report

### Case 1

**Fig. 1:**
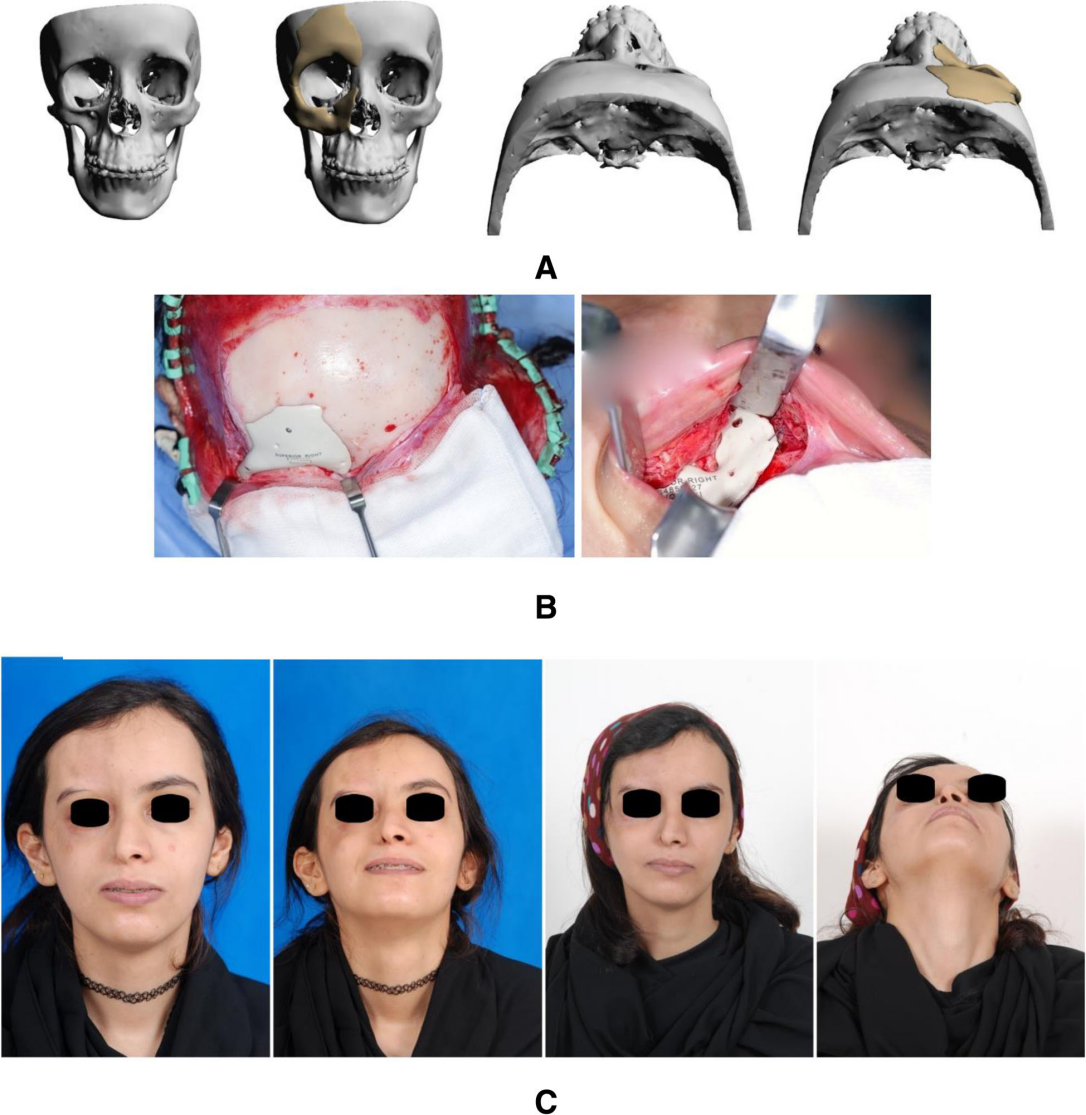
Parry-Romberg syndrome case. **a** 3D reconstruction of the CT showing the defects and the planned implants. **b** Intraoperative views of the implants. **c** Pre- and postoperative photographs of the patient

A 23-year-old female was diagnosed with Parry-Romberg syndrome, resulting in hypoplasia in the right side of her face. Patient had a history of fat grafting and fillers to cover the defect with unsatisfactory results. The patient underwent PEEK PSI reconstruction for her frontal bone, zygoma, and maxilla on the right side of her face through bicoronal and vestibular approaches. Patient satisfaction was reported as excellent postoperatively (Fig. 1).

### Case 2

**Fig. 2:**
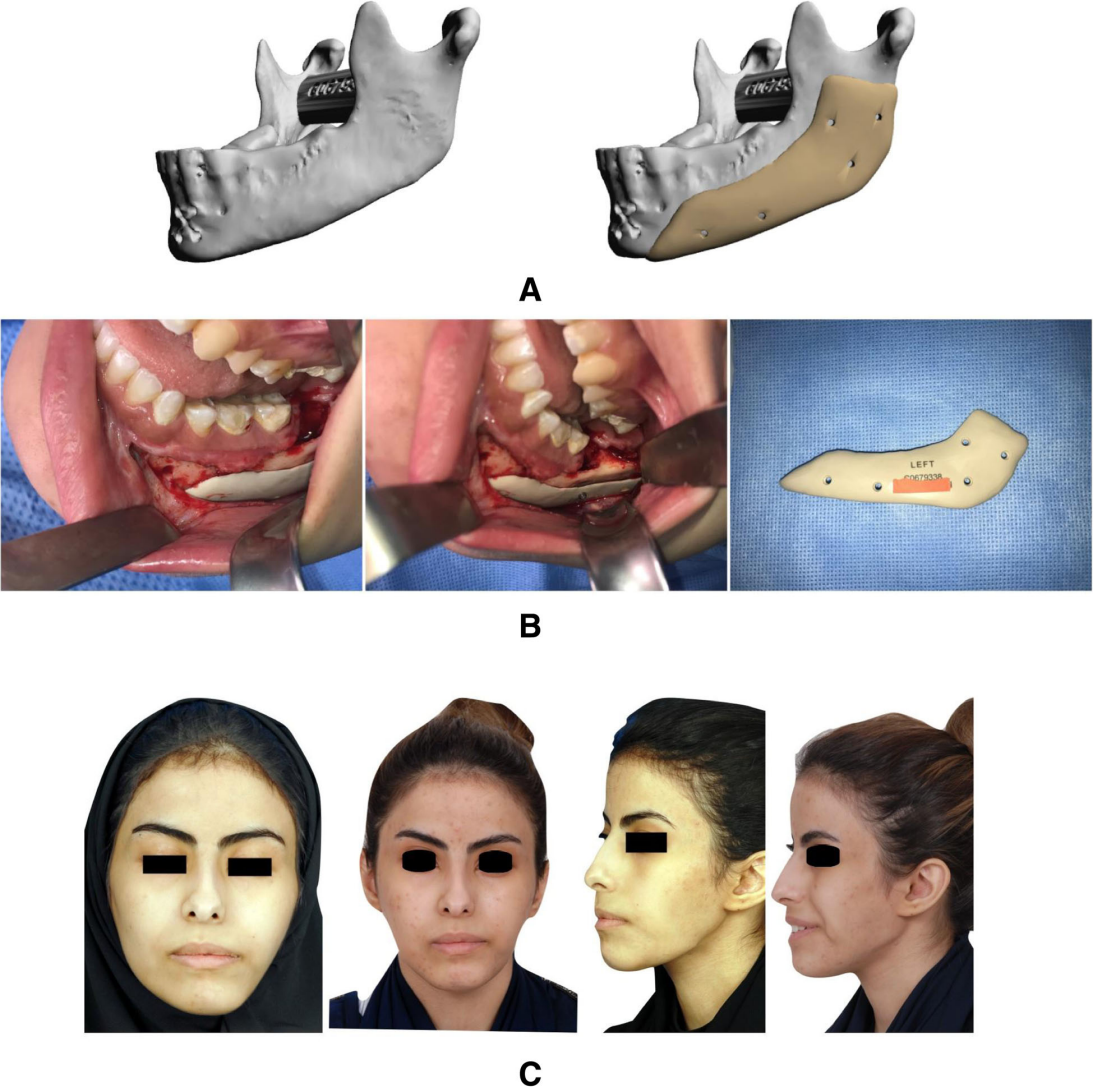
Hemifacial microsomia case. **a** 3D reconstruction of CT showing defect and planned implant. **b** Intraoperative view of the implant. **c** Pre- and postoperative photographs of the patient

A 28-year-old female was diagnosed with hemifacial microsomia, affecting her left side. She had a history of med-pore augmentation of the left body of mandible and ramus as well as genioplasty. However, the results were unfavorable in the angle area. The previous implant was removed and she underwent PEEK PSI reconstruction of the left body of mandible and ramus through a vestibular approach. Patient satisfaction was reported as excellent postoperatively (Fig. 2).

### Case 3

**Fig. 3:**
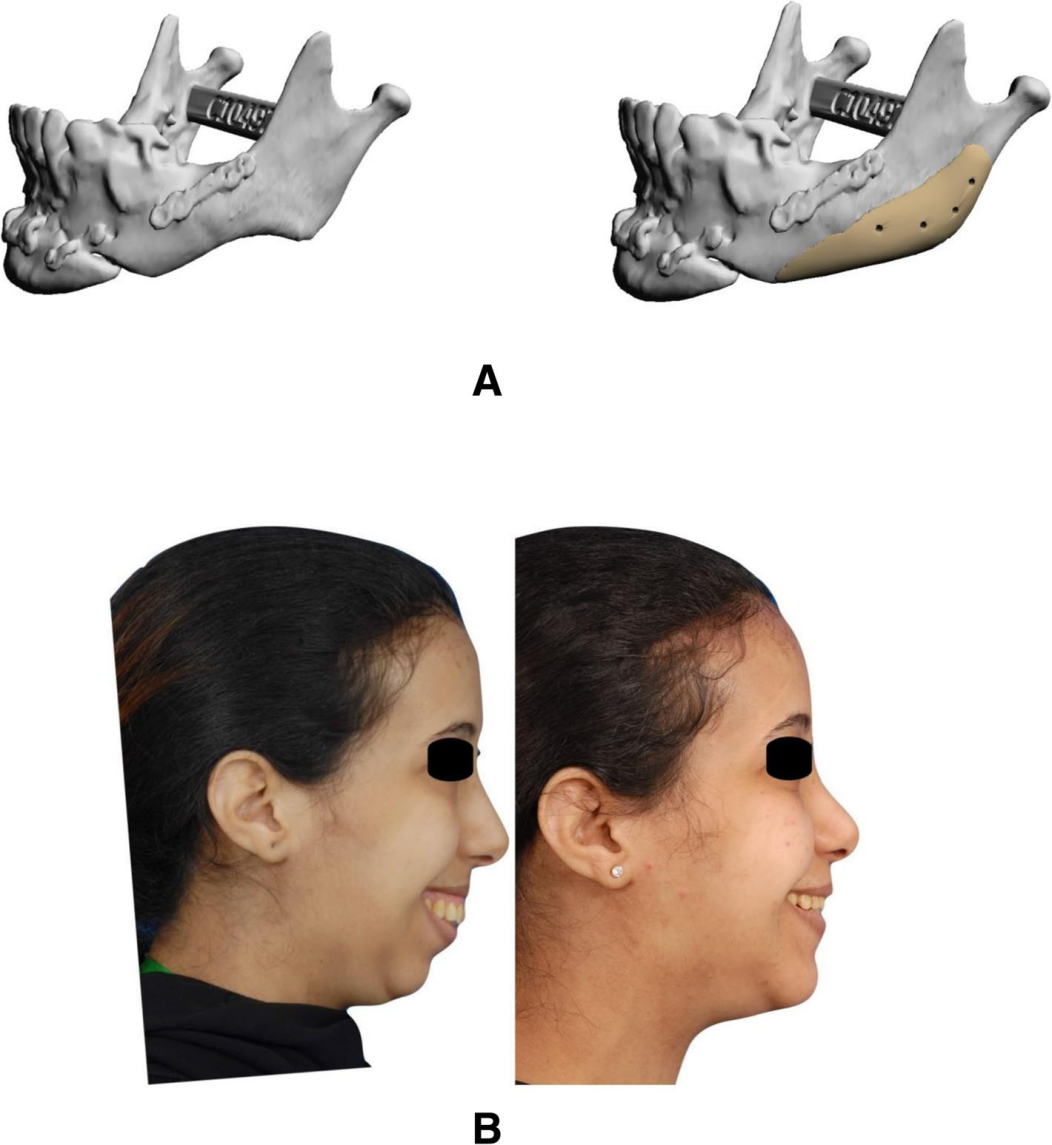
Post-BSSO case. **a** 3D reconstruction of CT showing defect and planned implants. **b** Pre- and postoperative photographs of the patient

A 38-year-old female underwent bilateral sagittal split osteotomy (BSSO) for a large mandibular advancement. Following the surgery, her mandibular angles were undefined bilaterally with deep antegonial angles. The patient underwent bilateral PEEK PSI reconstruction of her mandibular angles through a vestibular approach. Patient satisfaction was reported as excellent postoperatively (Fig. 3).

### Case 4

**Fig. 4:**
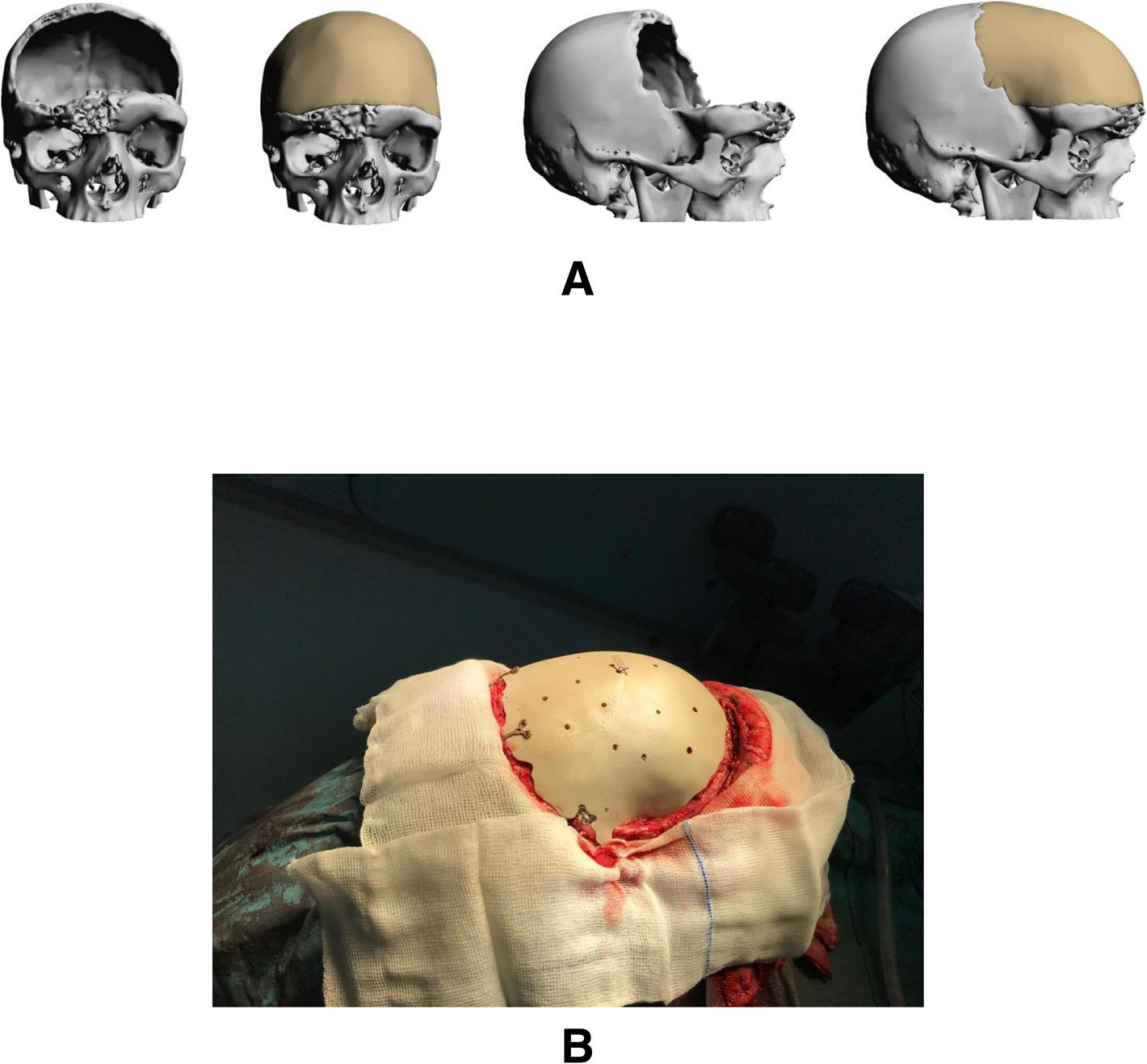
Craniotomy case. **a** 3D reconstruction of CT showing defect and planned implants. **b** Intraoperative views of the cranial implant

A 25-year-old male was seen at the neurosurgery department. Patient had a history of fibrous dysplasia affecting the frontal bone and cranium. He underwent multiple surgeries and reconstruction using titanium mesh. The result was deemed unsatisfactory. He was referred to our department for planning PEEK PSI for the cranium. A bicoronal approach was utilized for the surgery. Patient satisfaction was reported as excellent postoperatively (Fig. 4).

### Case 5

**Fig. 5:**
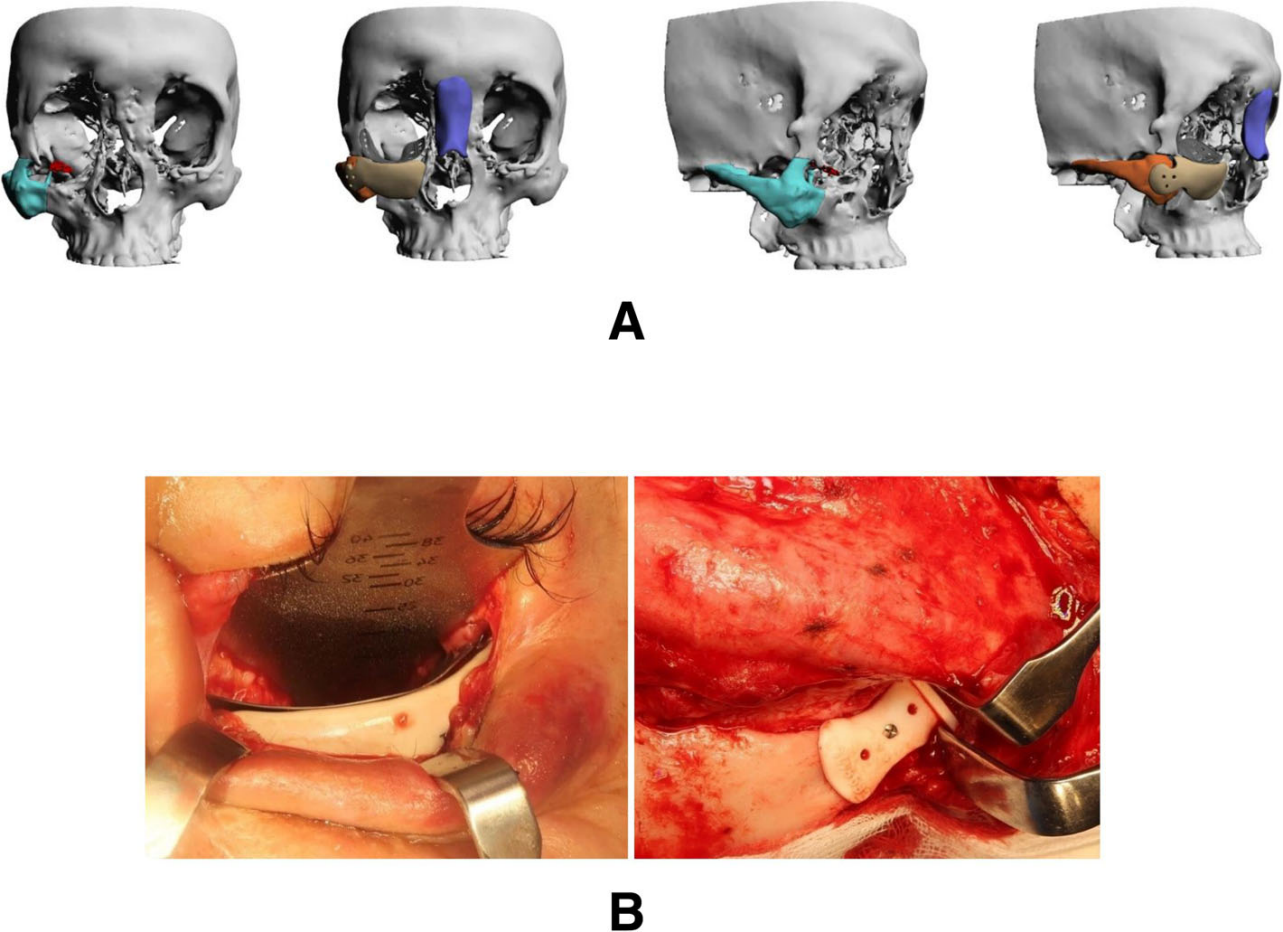
Post-traumatic defect case. **a** 3D reconstruction of CT showing defect, original position of zygoma and planned implants, and zygoma repositioning. **b** Intraoperative views of the implants

A 19-year-old male had a history of right zygomaticomaxillary complex fracture (ZMC). He presented complaining of secondary deformity as a result of facial trauma he sustained at the age of 16. He underwent zygomatic osteotomy and repositioning followed PEEK PSI reconstruction of right zygoma and nose as well as titanium PSI reconstruction of the right orbital floor. A bicoronal, transconjunctival, and vestibular approaches were used (Fig. 5).

### Case 6

**Fig. 6:**
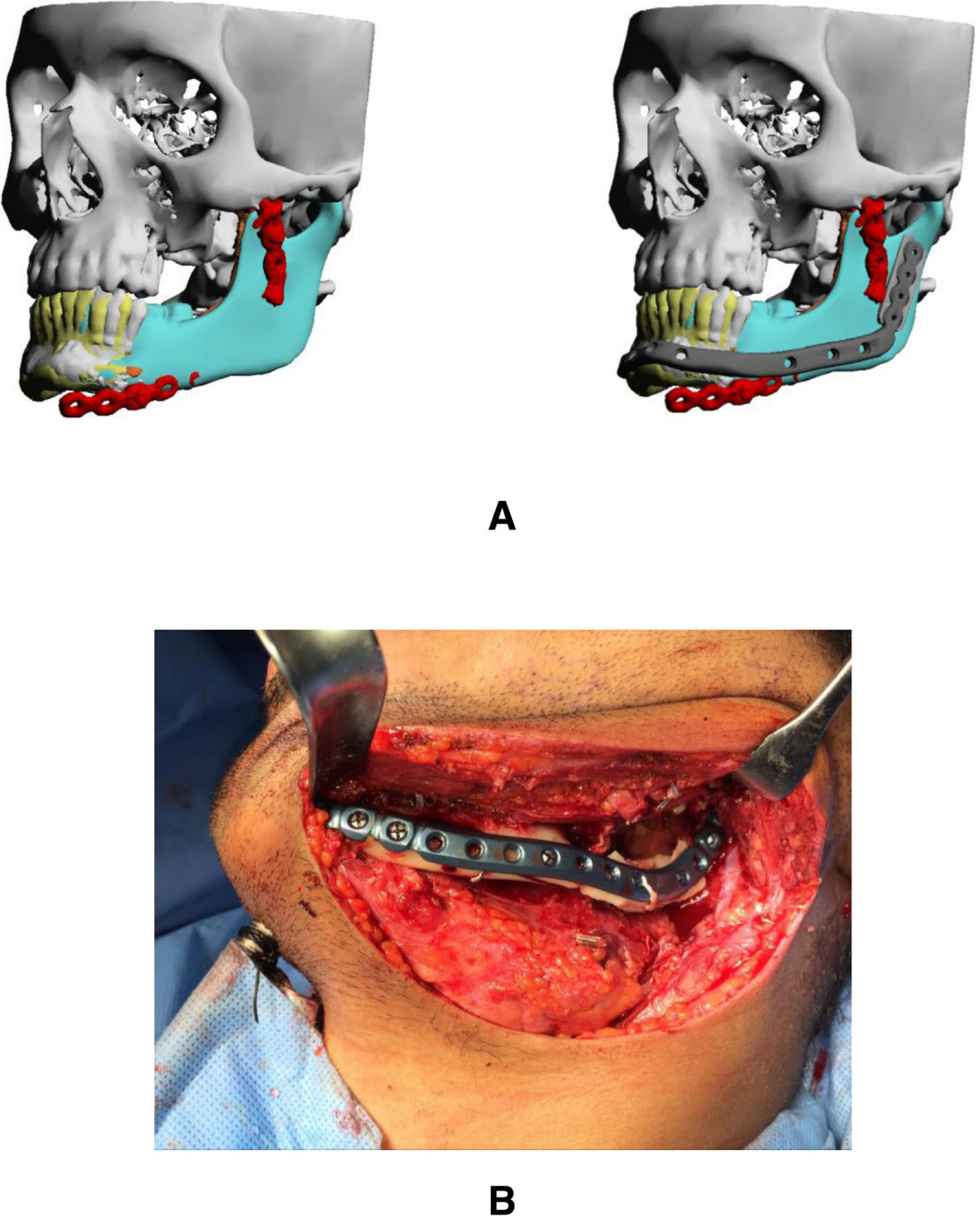
Post-free flap reconstruction case. **a** 3D reconstruction of CT showing previous plate and planned custom titanium implant. **b** Intraoperative view of the implant

A 50-year-old male was diagnosed with ameloblastoma affecting the left mandible. Patient underwent resection and reconstruction using a free fibula flap and titanium plate. The patient complained of an exposed plate following surgery. The plate was lost and dislocated from the condyle. A custom titanium plate was made to replace the exposed plate in his left mandible and was inserted through a submandibular approach. Patient satisfaction was reported as excellent postoperatively (Fig. 6).

No postoperative complications were seen in any of the patients; all recovered uneventfully. All patients stayed in the hospital for 1 day and were discharged the following day. No major complications were seen in the follow-up period. Patients were asked to report whether they were satisfied with the final result; all reported that they were satisfied from the functional and esthetic points of view. Table 1 summarizes the series results.

**Table 1:** Summary of all cases

Sex	Age	Etiology	Location	Approach	Adjustment	Satisfaction	Infection	Comments	Hospital stay (days)	Follow-up (months)
F	23	Rombreg syndrome	Frontal, zygoma, and maxilla	Bicoronal and vestibular	Trimming of nasal extension	Satisfied	None	Maxilla and zygoma implant was too big as 1 piece.	1	18
F	28	Hemifacial microsomia	Body and ramus of LT mandible	Vestibular	Designed to be away from mental nerve	Satisfied	None	None	1	11
F	34	Post-BSSO undefined jaw lines	Bilateral angle of mandible	Vestibular	None	Satisfied	None	Difficulty in screw fixation due to short implant	1	10
M	25	Post-craniotomy	Cranium	Bicoronal	Drainage holes created	Satisfied	None	None	1	10
M	19	Post-traumatic secondary deformity	Nasal ridge, zygoma, and orbit with cutting guides for zygoma repositioning	Bicoronal, transconjuctival, and vestibular	Trimming of nasal implant	Satisfied	None	Nasal implant was bulky.	1	12
M	50	Post-mandibular resection	Mandible	Submandibular	None	Satisfied	None	None	1	12

## Discussion

Maxillofacial defects are difficult to treat due to their important functional, esthetic, and psychological aspects. The anatomical complexity of this region has also contributed to the challenge it presents all surgeons, including veterans. Traditional pre-made implants often require many adjustments and usually offer suboptimal results [9]. The advances made in AM technology as well as 3D imaging have contributed greatly to the management of maxillofacial defects. This has facilitated the manufacturing of custom-made PSI that mirrors the healthy side to achieve a satisfactory result.

Computer-designed PSI offers higher accuracy and defect adaption, enhanced stability, more predictable outcomes, and better facial contour refinement [10]. Pre-made alloplastic implants usually require major intraoperative adjustments for large complex defects. In the literature, the usual complications associated with other materials, such as infection, foreign body reaction, and displacement, are seldom reported in relation to custom-made PSI [11]. This is in line with our experience in which none of the aforementioned complications occurred.

Using AM technology, we were able to operate on 6 patients with various maxillofacial defects using a total of 8 PEEKs and 2 titanium PSIs. We initially faced challenges meeting the requirements for completing proper preoperative CT for 3D planning. This was solved by always requesting 1–mm-thick CT for all PSI cases.

When designing custom-made PSI, engineers tend to design fixation screw holes in the area where the thickest bone is found irrespective of any vital tissues in the area. However, it is easy to drill wherever it is more preferable in the final implant regardless of the pre-designed screw hole. This is one of the advantages of using PEEK versus pre-made alloplastic implants such as silicone. It is not possible to drill a screw hole anywhere other than the pre-designed screw hole; doing so otherwise would lead to implant tearing and loss.

One of the challenges we faced was fixing mandibular angle PSI. In preformed alloplastic implants, the superior border is usually extended near the dentition to facilitate the fixation process. However, since PSI is based on mirroring of the healthy side, the superior portion is usually located near the inferior border of the mandible or middle part of the lateral cortex. We believe that this issue could be solved by planning all mandibular angle PSIs to have an extended superior border with a minimum thickness to facilitate the fixation process.

PEEK was used to fabricate 8 of the 10 PSIs used here. The use of PEEK in reconstructive surgery is well documented in the literature owing to its excellent biocompatibility, adjustability, stability, chemical inertness, radiolucency, and mechanical properties [12, 13].

In reconstructing the secondary deformity involving the zygoma and orbit, custom-made titanium implant was used in the orbit instead of PEEK because it is more affordable, and no further adjustments would be needed. The zygoma was reconstructed using a separate PEEK PSI.

In our experience, the PSI we used required minimal adjustments that were easily made intraoperatively. However, it should be noted that we faced issues inserting larger implants, requiring the extension of our surgical approach. We believe this could be easily alleviated by separating larger implants into separate smaller pieces with connectors in between as opposed to using 1 large piece.

A custom cutting guide was used in the post-traumatic secondary deformity case to design the zygomatic osteotomy. This was done to facilitate zygoma repositioning followed by PSI placement.

A major issue we faced was designing custom implants for the nasal area. This problem stemmed from the fact that it was impossible to mirror the healthy side since the entire bone was affected. To overcome this limitation, an average of healthy nasal bones was taken and implemented into the final design. However, the resulting implant was too bulky and required further intraoperative adjustments.

In all of our cases, we only reconstructed bony hard tissues. However, soft tissue evaluations were still necessary to ensure that optimum results were met. In the future, we believe that PSI designs should incorporate soft-tissue defects into it to plan PSI thickness accordingly.

None of our patients developed any complications related to the PSI reconstruction. The infection rate in our cases was 0%; wound healing was uneventful. In other reported cases, the infection rate following maxillofacial reconstruction PSI was low (7.7–14.3%) to nonexistent [14–16].

Our follow-up period was too short (mean, 9.4 months) to draw any long-term conclusions. However, the main concern following PSI reconstruction is postoperative infection [17]. No patient developed any signs of infection. Based on our long-term experience with non-custom-made implants such as silicone and porous polyethylene, postoperative infections are usually seen in the first few weeks and seldom seen soon after 1 month.

In our experience, the major drawback to the use of PSI is its high cost, which will surely drive many patients toward more affordable options. However, we believe the many advantages of using PSI outweigh this disadvantage.

## Conclusion

The use of PSI for maxillofacial reconstruction features predictable outcomes, eliminates the usual complications seen in non-custom-made implants, and boasts excellent patient satisfaction with high cost as the main drawback.

## Data Availability

All data and materials are available upon request.

## References

[1] Scolozzi P, Martinez A, Jaques B (2007). Complex orbito-fronto-temporal reconstruction using computer-designed PEEK implant. J Craniofac Surg.

[2] Tessier P (1982). Autogenous bone grafts taken from the calvarium for facial and cranial applications. Clin Plast Surg.

[3] Al-Ahmari A, Nasr EA, Moiduddin K, Alkindi M, Kamrani A Patient specific mandibular implant for maxillofacial surgery using additive manufacturing. In 2015 International Conference on Industrial Engineering and Operations Management (IEOM) (pp. 1-7). IEEE.

[4] Giannoudis PV, Dinopoulos H, Tsiridis E (2005). Bone substitutes: an update. Injury.

[5] Sbordone C, Toti P, Guidetti F, Califano L, Pannone G, Sbordone L (2014). Volumetric changes after sinus augmentation using blocks of autogenous iliac bone or freeze-dried allogeneic bone. a non-randomized study. J Craniomaxillofac Surg.

[6] Powell NB, Riley RW (1989). Facial contouring with outer-table calvarial bone: a 4-year experience. Arch Otolaryngol Head Neck Surg.

[7] Honigmann P, Sharma N, Okolo B, Popp U, Msallem B, Thieringer FM (2018) Patient-specific surgical implants made of 3D printed PEEK: material, technology, and scope of surgical application. BioMed Res Int 10.1155/2018/4520636 PMC5884234 29713642

[8] Rana M, Chui CH, Wagner M, Zimmerer R, Rana M, Gellrich NC (2015). Increasing the accuracy of orbital reconstruction with selective laser-melted patient-specific implants combined with intraoperative navigation. J Oral Maxillofac Surg.

[9] Kim MM, Boahene KD, Byrne PJ (2009). Use of customized polyetheretherketone (PEEK) implants in the reconstruction of complex maxillofacial defects. Arch Facial Plast Surg.

[10] Owusu JA, Boahene K (2015). Update of patient-specific maxillofacial implant. Curr Opin Otolaryngol Head Neck Surg.

[11] Binder WJ, Kaye A (1994). Reconstruction of posttraumatic and congenital facial deformities with three-dimensional computer-assisted custom designed implants. Plast Reconstr Surg.

[12] Järvinen S, Suojanen J, Kormi E, Wilkman T, Kiukkonen A, Leikola J, Stoor P (2019). The use of patient specific polyetheretherketone implants for reconstruction of maxillofacial deformities. J Craniomaxillofac Surg.

[13] Kurtz SM (2012) An overview of PEEK biomaterials. In: PEEK biomaterials handbook. William Andrew Publishing:1

[14] Nieminen T, Kallela I, Wuolijoki E, Kainulainen H, Hiidenheimo I, Rantala I (2008). Amorphous and crystalline polyetheretherketone: mechanical properties and tissue reactions during a 3-year follow-up. J Biomed Mater Res A.

[15] Scolozzi P (2012). Maxillofacial reconstruction using polyetheretherketone patient-specific implants by “mirroring” computational planning. Aesthet Plast Surg.

[16] Alonso-Rodriguez E, Cebrián JL, Nieto MJ, Del Castillo JL, Hernández-Godoy J, Burgueño M (2015). Polyetheretherketone custom-made implants for craniofacial defects: report of 14 cases and review of the literature. J Craniomaxillofac Surg.

[17] Rosenthal G, Ng I, Moscovici S, Lee KK, Lay T, Martin C, Manley GT (2014). Polyetheretherketone implants for the repair of large cranial defects: a 3-center experience. Neurosurgery.

